# Short-term effects of remote ischemic conditioning on vascular function in patients with cardiovascular diseases: a systematic review with meta-analysis

**DOI:** 10.31744/einstein_journal/2025RW1937

**Published:** 2025-08-12

**Authors:** Samuel Amorim, Gilberto Laurentino, Victor Sabino de Queiros, Yujia Liu, Per Aagaard, Charlotte Suetta, Naufal Zagidullin, Kenneth J. Gollob

**Affiliations:** 1 Hospital Israelita Albert Einstein São Paulo SP Brazil Hospital Israelita Albert Einstein, São Paulo, SP, Brazil.; 2 Universidade São Judas Tadeu São Paulo SP Brazil Universidade São Judas Tadeu, São Paulo, SP, Brazil.; 3 Universidade Federal do Rio Grande do Norte Natal RN Brazil Universidade Federal do Rio Grande do Norte, Natal, RN, Brazil.; 4 Jiangsu Normal University Department of Physical Education Xuzhou Jiangsu China Department of Physical Education, Jiangsu Normal University, Xuzhou, Jiangsu, China.; 5 University of Southern Denmark Department of Sports Science and Clinical Biomechanics Odense Denmark Department of Sports Science and Clinical Biomechanics, University of Southern Denmark, Odense, Region Syddanmark, Denmark.; 6 University of Copenhagen Department of Geriatrics Copenhagen Capital Region of Denmark Denmark Department of Geriatrics, University of Copenhagen, Copenhagen, Capital Region of Denmark, Denmark.; 7 Bashkir State Medical University Department of Internal Diseases Ufa Republic of Bashkortostan Russian Federation Department of Internal Diseases, Bashkir State Medical University, Ufa, Republic of Bashkortostan, Russian Federation.

**Keywords:** Cardiovascular diseases, Cardiovascular system, Vascular stiffness, Ischemia, Carotid-femoral pulse wave velocity, Endothelium

## Abstract

**Objective:**

The purpose of this systematic review with meta-analysis was to compare the short-term (≤1 month) effects of remote ischemic conditioning *versus* sham remote ischemic conditioning on vascular function in patients with cardiovascular diseases.

**Methods:**

A systematic review was conducted to identify relevant studies through six healthcare science databases up to March 2025. Arterial stiffness and endothelial function were defined as the primary outcome. Meta-analyses were performed using a random-effect model. Also, GRADE Pro software was used to evaluate the quality of evidence.

**Results:**

A total of 7 randomized clinical trials with 603 patients with cardiovascular diseases were included in the systematic review. Also, we included 5 studies in the meta-analysis. Compared with sham remote ischemic conditioning or control, short-term remote ischemic conditioning resulted in enhanced endothelial function (MD=4.22% [95%CI=1.75; 6.69]; p=0.0008; I^2^=0%) without any changes in arterial stiffness, (MD=-0.05 m/s [95%CI =-0.77, 0.67]; p=0.89; I^2^=89%).

**Conclusion:**

This systematic review and meta-analysis found that short-term remote ischemic conditioning was associated with improvements in endothelial function compared with sham remote ischemic conditioning or control in cardiovascular patients, without any impact on arterial stiffness.

**Prospero database registration:**

ID CRD42021234702.

## INTRODUCTION

Remote ischemic conditioning (RIC) involves repeated bouts of brief ischemia followed by transient reperfusion, which induces tissue resistance against free radical damage from subsequent ischaemic insults in distant tissues, that may confer protection to other organs, including the heart.^(1)^ RIC activates multiple signalling pathways that promote cellular survival and reduce cell death.^(2)^ Remote ischemic conditioning can modulate the inflammatory response following acute ischemia-reperfusion injury by reducing the release of pro-inflammatory cytokines and chemokines that promotes anti-inflammatory mediators.^(3)^ By attenuating the magnitude of local inflammation, RIC limits tissue damage and promotes tissue repair.^(3)^ Moreover, RIC induces the release of various substances from the remote organ, such as adenosine, opioids, and bradykinin, that have protective effects on the heart tissue^(4)^ by activating endogenous cytoprotective pathways, such as the opening of mitochondrial ATP-sensitive potassium channels, which reduce cellular injury.^(5)^ Also, RIC may facilitate the expression of stress response proteins, including heat shock proteins (HSPs), which also are known to have cytoprotective effects.^(6)^ Heat shock proteins help to maintain protein folding, prevent protein aggregation, and promote cell survival under stressful conditions.^(7)^ Then, RIC-induced miRNAs have been shown to modulate several signalling pathways involved in cardioprotection.^(8)^

Murry^(9)^ was the first to demonstrate that repeated bouts of ischemia and reperfusion in coronary arteries could protect against subsequent myocardial and vascular damage, causing marked reductions in infarct size, as verified by later studies.^(10,11)^

In canine hearts that received brief periods of circumflex artery occlusion and reperfusion, subsequent left anterior descending territory infarctions were smaller than in control hearts.^(12)^ Specifically, RIC reduced infarct size from 60±5 to 35±5% and reduced the area at risk from 57±7 to 27±3% in isolated rabbits’ heart.^(13)^ In humans, RIC appears to reduce the magnitude of acute myocardial infarction and possibly improve myocardial salvage before percutaneous coronary intervention.^(14,15)^ Further, RIC may induce a decreased release of troponin in patients undergoing coronary artery bypass surgery^(16)^ thereby reducing the incidence of periprocedural myocardial infarction, particularly when used in the lower body and for patients with multivessel disorders.^(17)^ Also, previous studies have suggested that brief, remote ischemia-reperfusion cycles may elicit cardioprotection against acute ischemic insults, mediated by the release of humoral and neural protective factors into the systemic circulation.^(18,19)^

As arterial stiffness increases or decreases with ageing or endurance exercise, respectively, accompanied by distinct functional and structural changes in the vascular wall.^(20)^ Increased arterial stiffness represents the main age-related risk factor for systolic hypertension^(21)^ and cardiovascular diseases.^(22)^ In addition, elevated blood pressure is positively correlated to increased arterial stiffness in young adults,^(23)^ while also associated with an increased incidence of heart failure in middle-aged and older adults.^(24)^ In parallel, these changes can be related to signs of endothelial dysfunction that causes impairments in the peripheral delivery and exchange of oxygen/nutrients, dysregulation of blood flow dynamics and haemostasis, and gives rise to imbalances in tissue-blood barrier functions and tissue-specific angiocrine signaling pathways,^(25,26)^ respectively.

The short-term effect of RIC on arterial stiffness and endothelial function remains unknown, notably also in clinical populations.

## OBJECTIVE

Therefore, the aim of this systematic review and meta-analysis was to evaluate the effects of short-term (≤1 month) Remote ischemic conditioning intervention on arterial stiffness and endothelial function in patients with cardiovascular diseases.

## METHODS

This systematic review with meta-analysis was performed in accordance with the Preferred Reporting Items for Systematic Reviews and Meta-Analyses (PRISMA) guidelines.^(27)^

### Search strategy

Studies were retrieved through a systematic literature search in MEDLINE via Pubmed, EMBASE via Ovid, CINAHL (including pre CINAHL) via EBSCO, Web of Science, Sports Discus via EBSCO and the Cochrane Central Register of Controlled Trials up to August 2023. The major search terms were (all databases): ("Ischemic Preconditioning"[Mesh] OR (ischaemic OR ischemic OR ischaemia OR ischemia) AND (preconditioning OR preconditionings OR pre-conditioning OR pre-conditionings OR pre conditioning OR pre conditionings) AND "remote ischemic conditioning", "remote ischemic preconditioning", "ischemic preconditioning", ("vascular stiffness"[Mesh] OR "arterial stiffness" OR "aortic stiffness") AND (Pulse wave analysis"[Mesh] OR "aortic pulse wave velocity") AND ("carotid-femoral pulse wave velocity"[Mesh] OR "carotid-femoral pulse wave velocities") OR endothelium" [Mesh] OR "vascular endothelium", OR " flow mediated dilation") AND (cardiovascular diseases" OR "heart disease" OR vascular disease" OR "cardiovascular abnormalities"). In addition, only studies published in English language were included in the present analysis.

### Eligibility criteria

Only randomized controlled clinical trials were considered eligible. There was no restriction by year of publication, but only studies published in English language were included. The following eligibility criteria were adopted for the studies selection:

Population: Adults (≥18-year-old) with clinically verified cardiovascular disease; Intervention: Short-term RIC (≤1-month only exposure to RIC, including single bout or repeated sessions of RIC); Comparator: SHAM treatment or passive control; Outcomes: Arterial stiffness and endothelial function, based on recordings of aortic pulse wave velocity (PWV) and brachial or femoral flow-mediated dilation (FMD) (gold standard methods to evaluate vascular function).^(28,29)^ Additionally, these outcomes were included separately providing the overall function of the vascular system.

### Data source and search strategy

Studies were retrieved through a systematic literature search in MEDLINE (via PubMed^®^), EMBASE (via Ovid), Cochrane Central Register of Controlled Trials, Cumulative Index to Nursing and Allied Health Science (CINAHL), including pre CINAHL (via EBSCO), SPORTDiscus (via EBSCO), and Web of Science up to August 2024.

### Study selection and data extraction

The selection of studies was performed by two researchers independently. Conflicts about study eligibility were resolved by a third researcher. The selection of studies was divided into two stages. In the first stage, studies were selected by reading titles and abstracts. Subsequently, the studies were selected through the complete reading of the study (second stage).

Data from included articles were extracted utilizing a data extraction form comprising: medium and standard deviation pre/post intervention, sample size, study design, clinical population characteristics (age, sex, cardiovascular conditions or diseases), ischemic time, protocol used, cuff pressure and main results.

### Risk of bias

Cochrane's risk of bias tool was used to assess potential bias of the included studies. ^(30)^ The risk of bias assessment scores on reporting of judgment items were: (i) Adequate (A risk of bias that will not have a significant impact on the results), (i) Unclear (Bias that may have a significant impact on the results), and (iii) Inadequate (Bias that might have had a negative impact on the results). Each study was assessed individually based on seven explicit criteria by the principal author. Also, attrition bias and reporting bias were also considered, as well as selection bias, performance bias, and detection bias.

### Effect measures and synthesis methods

ΔSD were calculated for conditions RIC and control/SHAM as: ΔS=√(SD_pre^2 +SD_(post)^2×2 ×0.5 ×SD_(pre)×SD post).^(31)^ For studies with multiple arms, ΔMean and ΔSD were calculated independently for each condition and subsequently combined using the Review Manager (RevMan, Version 5.3, The Cochrane Collaboration, 2014). The Δmean and the ΔSD were used to calculate the mean difference (MD) and the standard error (SE) and, posteriorly, pooled using the generic inverse variance method. Random effects meta-analyses were performed in RevMan (Version 5.3, The Cochrane Collaboration, 2014). The statistical heterogeneity of treatment effects between studies was assessed using the I^2^ inconsistency test.^(32)^ Inconsistency was classified as: low (<25%), moderate (25-49%) and high (>50%). It was not possible to analyze publication bias due to the low number of studies included in the analyzes (<10).^(33)^

### Quality assessment

GRADE methodology was used to assess the quality of the retrieved evidence (GRADEpro, Version 20. McMaster University, 2014). According to GRADE, randomized clinical trials are high-quality studies (score 4) but can be reduced in quality according to the identified bias risks, such as moderate, low, or very low.^(34)^ In this evaluation, we examined the following topics: (i) methodological limitations identified in the studies (risk of bias), (ii) inconsistency in results (heterogeneity), (iii) indirect evidence, (iv) imprecision, and (v) publication bias. A two-factor assessment was conducted to determine whether indirect evidence was present: (1) when interventions did not match the desired intervention and (2) when substitute results were substituted instead of relevant results. In cases of imprecision, the evidence was downgraded when a wide 95% confidence interval (95%CI) was identified, or a small number of studies was selected for the meta-analysis (≤5).

## RESULTS

### Study selection

**Figure 1:**
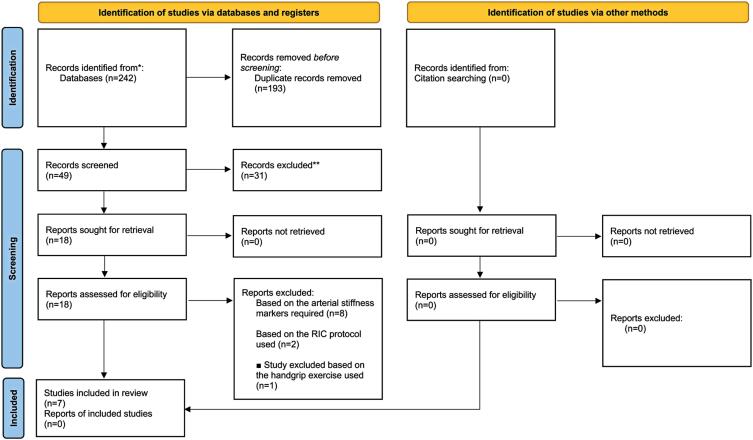
Flow chart of study selection process

The present systematic search identified a total of 242 studies. Of these, 49 studies were selected for full-text reading; of these, seven studies fulfilled the eligibility criteria, including high methodological quality (Figure 1).

### Study characteristics

All included studies used (a) randomized participant allocation procedures, (b) involved short-term (≤1 month) RIC intervention protocol.

### Participants

A total of seven studies were included, involving a total of 603 patients with cardiovascular diseases, aged 52 to 67-years. Two studies included patients with acute myocardial infarction.^(35,36)^ A single study included patients with angina pectoris,^(37)^ while the remaining studies included patients with coronary heart disease,^(38)^ stroke survivors,^(39)^ patients with vascular and cardiovascular disorders^(40)^ and patients undergoing vascular surgery.^(41)^

### Outcome variables

A systematic assessment of changes caused by RIC intervention on arterial stiffness and endothelial function markers in patients with cardiovascular diseases was performed (Tables 1 and 2). Arterial stiffness and endothelial function were evaluated using applanation tonometry (PWV) and ultrasonography (FMD), respectively.

**Table 1:** Short-term effects of remote ischemic conditioning on pulse wave velocity in patients with vascular and cardiovascular diseases

Authors	Subjects	Trial design	Protocol used	Outcomes and main results
Ikonomidis et al.^(35)^	RIC single-cycle: n=90 Age: 53±16 RIC double-cycle: n=90 Age: 54±16 Control: n=90 Age:52±16 Population: PWSTEMI	Randomized parallel trial Researchers blinded	1-2 cycles of 5 min of ischemia with 5 min of reperfusion between cycles Where used: Upper arms Period: Once after primary PCI Cuff pressure used (mmHg): RIC:200	The primary outcome was carotid femoral pulse wave velocity (PWV) RIC groups decreased significantly PWV RIC single-cycle (PWV – m/s) PWV (12.09±0.6 pre to 11.71±0.65 post) RIC double-cycle (PWV – m/s) PWV (12.06±0.5 pre to 13.8±0.7 post) Control (PWV – m/s) PWV (11.7±0.8 pre to 11.7±1.5)
Kepler et al.^(40)^	RIC: n=44 Age: 67±9 Sham RIC: n=46 Age: 66±10 Population: PUVS	Randomized parallel trial Double-blinded	4 cycles of 5 min of ischemia with 5 min of reperfusion between cycles Where used: Upper arm Period: Once before aneasthesia Cuff pressure used (mmHg): RIC:200 / Sham:10-20	The primary outcome was carotid femoral pulse wave velocity (PWV) RIC has no effect on PWV RIC: PWV mean change −0.26 m/s (SD 1.68) Sham RIC: PWV mean change −0.4 m/s (SD 1.38)
Kuusik et al.^(41)^	RIC: n=47 (33 M / 14W) Age: 66.1±10.2 Sham RIC: n=55 (48M / 7W) Age: 65.1±11.4 Population: PAD	Randomized parallel trial Double-blinded	4 cycles of 5 min of ischemia with 5 min of reperfusion between cycles Where used: Upper arm Period: Once before the subsequent angiographic Cuff pressure used (mmHg): RIC:200 / Sham:20	The primary outcome was carotid femoral pulse wave velocity (PWV) RIC has no effect on PWV RIC (PWV: 5.18±0.37 pre to 5.21±0.41 post) Sham RIC (PWV: 5.21±0.43 pre to 5.20±0.45 post)
Zagidullin et al.^(37)^	RIC HI: n=20 (16M/ 4W) Age: 58±2 RIC PWSAP: n=30 (21M / 9W) Age: 63±1 Population: PWSAP and HI	Randomized crossover	3 cycles of 5 min of ischemia with 5 min of reperfusion between cycles Where used: Forearm Period: Once before PCI Cuff pressure used (mmHg): 50 mmHg up to from SBP	The primary outcome was carotid femoral pulse wave velocity (PWV) RIC has no effect on PWV RIC (5.37±0.8 pre to 4.95±0.49 post – Delta: 0.42±0.53 Sham RIC: (5.5±0.6 pre to 6.0±0.63 post – Delta: 0.5±0.3

RIC: remote ischemic conditioning: PAD: patients with symptomatic peripheral arterial disease; PWSAP: patients with stable angina pectoris; PUVS: patients undergoing vascular surgery; PWAPD: patients with arterial peripheral diseases; W: women; M: male; PWSTEMI: patients with ST-elevation myocardial infarction; HI: healthy individuals; PCI: percutaneous coronary intervention.

**Table 2:** Short-term effects of ischemic conditioning on endothelial function in patients with cardiovascular diseases

Authors	Subjects	Trial design	Protocol used	Outcomes and main results
Hyngstrom et al.^(39)^	RIC: n=12 Age: 60±16 Sham RIC: n=11 Age: 60±18 Population: Stroke survivors	Randomized parallel trial Subjects blinded	5 cycles of 5 min of ischemia with 5 min of reperfusion between cycles Where used: Affected thigh Period: Every other day for 2 weeks Cuff pressure used (mmHg): RIC:225 / Sham:10	The primary outcome was Brachial Artery FMD Assessment RIC group increased significantly Brachial Artery FMD RIC (5.4±4.7% pre to 7.8±4.4 % post; p=0.030) RIC Sham (3.5±3.9% pre to 2.4±3.1 post; p=0.281)
Liang et al.^(38)^	RIC: n=20 (12 M / 8W) Age: 64±10 Control (PWBC): n=20 (11M / 9W) Age: 64±10 PCHD: n= 20 (8M / 12W) Age: 64±10 Population: PCHD	Randomized parallel trial	4 cycles of 5 min of ischemia with 5 min of reperfusion between cycles Where used: Upper arm Period: 3 times a day for 20 days Cuff pressure used (mmHg): RIC:200	The primary outcome was Brachial Artery FMD Assessment FMD was improved in the RIC group compared to the CHD group RIC (5.5%±3.3 pre to 8.5%±2.4 post) PCHD (4.6%±3.2 pre to 4.9%±4.2 post)
Manchurov et al.^(36)^	RIC: n=23 (12M / 11W) Age: 63 Control: n=25 (14M /11W) Age: 61	Randomized parallel trial	4 cycles of 5 min of ischemia with 5 min of reperfusion between cycles Where used: Upper limbs Period: Once before PCI Cuff pressure used (mmHg): RIC:200	The primary outcome was Brachial Artery FMD Assessment FMD tests showed significantly higher RIC than control at day 7 RIC (5.9% pre to 12.3% post) Control (7.1% pre to 7.5% post)

RIC: remote ischemic conditioning: W: women; M: male; PAMI patients with acute myocardial infarction; PCI: percutaneous coronary intervention; PCHD: Patients with coronary heart disease; PWBC: Patients with breast cancer.

### Risk of bias of individual studies

**Figure 2:**
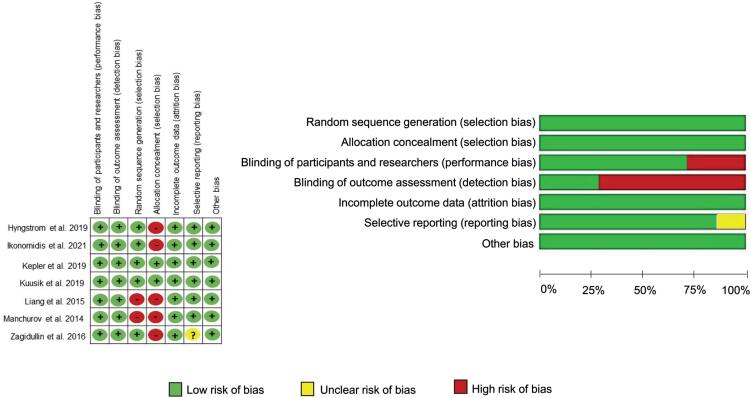
Summary of risk assessment of bias of included articles and risk of bias graph presented as a percentage of all items included

Risk of bias for each included study is presented in figure 2, with data summarized as a percentage across all included studies. The blinding of outcome assessment and the blinding of participants was found to pose high risks of bias in five studies^(35-39)^ and two studies, respectively.^(36,42)^ Additionally, a single study reported unclear bias in the selective reporting item.^(37)^ The studies included in our analysis had an overall low bias.

### Quantitative analyses (Meta-analysis)

**Figure 3:**
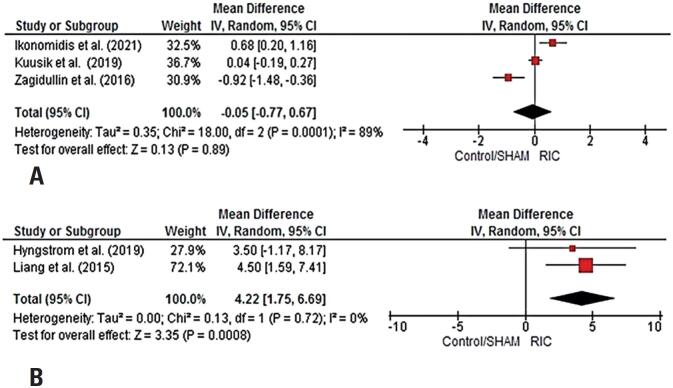
A) Meta-analysis of the effects of RIC on arterial stiffness in patients with cardiovascular diseases. B) Meta-analysis of the effects of RIC on endothelial function in patients with cardiovascular diseases. The solid red square represents study-specific estimates, and the solid diamond represents pooled estimates of random-effects

Three studies (3 comparisons) were included in the comparisons between RIC versus Control/SHAM on arterial stiffness (PWV). One study was not included in this comparison due to data distribution and reported measure of central tendency and dispersion (Median and interquartile range).^(40)^ The analysis showed no differences between interventions, but a high level of inconsistency (MD=-0.05 m/s [95%CI=-0.77, 0.67]; p=0.89; I^2^=89%) (Figure 3A).

95%CI: 95% confidence interval.

Two studies were included in comparisons in FMD between RIC versus Control/SHAM. One study could not be included due to data distribution and measured central tendency (median and interquartile range).^(36)^ The results were in favor of RIC treatment (MD=4.22% [95%CI=1.75; 6.69]; p=0.0008; I^2^=0%) (Figure 3B).

### Study quality assessment

The included studies examining arterial stiffness and endothelial function all could be classified as high and moderate quality using the GRADEpro tool (Table 3). Although not observed in the present meta-analysis, a low quality of studies typically is associated with the lack of specifications for blinding participants and assessors. Randomized controlled trial quality, therefore, may be enhanced by focusing on these aspects in future intervention studies exploring RIC.

**Table 3:** GRADEpro assessment

Certainty assessment							№ of patients		Effect		Certainty	Importance
№ of studies	Study design	Risk of bias	Inconsistency	Indirectness	Imprecision	Other considerations	RIPC	Sham	Relative (95% CI)	Absolute (95% CI)		
Aortic pulse wave velocity (follow-up: mean 1 days; assessed with: Applanation tonometry)												
3	Randomized trials	Not serious	Not serious	Not serious	Not serious	None	211	221	-	MD 0.05 m/s fewer (-0.77 fewer to 0.67 more)	⊕⊕⊕⊕ High	Important
Flow-mediated dilation (follow-up: range 1 days to 21 days; assessed with: Ultrasound)												
2	Randomized trials	Serious^a^	Not serious	Not serious	Not serious	Strong association	32	30	-	MD 4.22 % more (1.75 more to 6.69 more)	⊕⊕⊕○ Moderate^a^	Important

95% CI: confidence interval; MD: mean difference.

Explanations

a One study did not blind outcome data and participants.

## DISCUSSION

The aim of the present study was to review the literature and conduct a meta-analysis to verify potential differences between RIC and control or sham RIC on arterial stiffness and endothelial function. Our analysis indicated that RIC could induce positive effects on endothelial function in patients with cardiovascular disease, while in contrast not effective in reducing arterial stiffness.

### Arterial stiffness

The present meta-analysis revealed RIC to have no short-term effect on carotid to radial pulse wave velocity (PWV) in patients with cardiovascular disorders compared to control conditions or sham RIC. In support of this observation, it has previously been shown that two sessions of RIC did not have any acute impact on PWV in healthy young people.^(43)^ Acute hyperaemia induced by prolonged ischemia and reperfusion in the ipsilateral limb normally decreases PWV, however RIC performed in contralateral limb may prevent this blunting response in young healthy male subjects.^(44)^ The decline in PWV caused by acute hyperaemia appears to be regulated by endothelial mechanisms.^(45)^ Additionally, greater PWV increases myocardial workload by increasing the reflection wave from the periphery, augmenting left ventricular afterload and thereby potentially reducing coronary perfusion.^(46)^ In addition, increases in PWV have been linked to age independent changes in risk factors for atherosclerosis and other risk factors.^(47)^

The present observations indicate that short-term administration of RIC has no major impact on PWV in patients with cardiovascular diseases. Importantly, the participants included in the present studies did not report any side effects using RIC, indicating that the method may be feasible and safe in patients with cardiovascular diseases.

### Endothelial function

Several cardiovascular outcomes are predicted by endothelial dysfunction in the atherosclerotic cascade.^(48)^ Furthermore, endothelial dysfunction induced by ischemia-reperfusion is generally prevented with RIC.^(49,50)^ The improvement in conduit artery function can be attributed to elevated shear stresses as a major physiological stimulus.^(51)^ More remote areas, however, have relatively low shear levels during RIC exposure.^(52)^ In healthy humans, RIC improves local and systemic endothelial function acutely and these effects persist one week later.^(52)^ Notably, the present meta-analysis demonstrates that RIC may be effective of enhancing endothelial function compared to passive control conditions or sham RIC.

**Figure 4:**
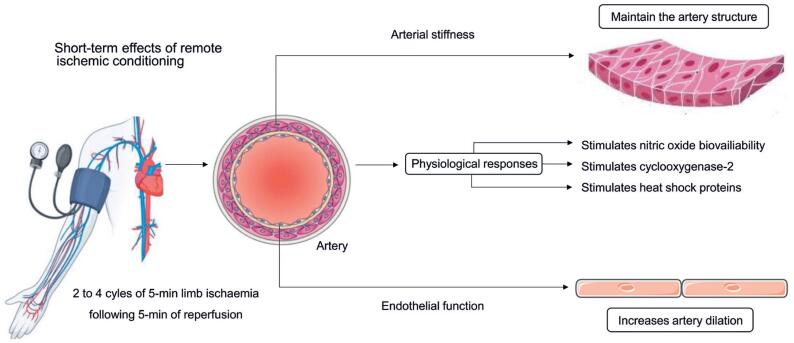
A possible physiological mechanism underlying RIC's effects on vascular function in patients with cardiovascular diseases

Vascular endothelial growth factor (VEGF) and endothelial progenitor cells are increased in response to RIC, which have been suggested to enhance endothelial function.^(53-55)^ The release of endothelial progenitor cells from bone marrow is known to be stimulated by tissue ischemia^(56)^ and shear stress,^(57)^ which are both present during and following acute RIC. In addition, RIC may reduce oxidative stress by improving antioxidative defence mechanisms (*e.g.*, increasing superoxide dismutase activity) and/or lowering oxygen free radical levels.^(58,59)^ Also, adenosine, bradykinin, norepinephrine and opioids are known to be released from the capillary walls during ischemic conditions,^(60-62)^ which may prevent damage by activation of potassium channels and inducing increased intracellular ATP stores.^(63)^ This protective mechanism may also involve increased bioavailability of nitric oxide (NO), cyclooxygenase-2 (COX-2), and heat shock proteins (HSPs)^(19,64)^ that together may contribute to enhance femoral flow-mediated dilation (FMD) in conduit arteries (Figure 4).

### Limitations

This systematic review and meta-analysis are not without limitations. First, we only evaluated the short-term effects (≤1 month) of RIC on arterial stiffness and endothelial function in patients with cardiovascular diseases. Hence, the effect of long-term RIC application on markers of arterial stiffness and endothelial function in this specific population remains unknown. Second, the influence of other factors related to the specific RIC protocol, such as the RIC exposure duration, number of cycles, pneumatic pressure applied and duration of ischemia/reperfusion, and combinations thereof were not assessed in the current meta-analysis.

## CONCLUSION

### Perspectives for cardiovascular rehabilitation

The present systematic review and meta-analysis suggests that short-term remote ischemic conditioning has no negative (nor positive) impact on arterial stiffness in patients with cardiovascular diseases. The potential clinical benefits of remote ischemic conditioning, however, appears to be its positive effects on endothelial function in these patients, as revealed by the present meta-analysis. The present observations do not exclude that detrimental effects of remote ischemic conditioning on arterial stiffness and endothelial function may be present in other clinical populations (frail older adults with arterial hypertension, patients with coronary heart disease, heart failure with preserved or reduced ejections fraction), which warrants evaluation in future studies. Moreover, the potential physiologic mechanisms behind the remote ischemic conditioning induced changes in endothelial function warrant further study and could lead to identification of potential biomarkers of response intensity, as well as explain why some patients respond better than others. Hence, remote ischemic conditioning studies should be performed to verify the efficacy of remote ischemic conditioning to improve vascular function and its safety with more prolonged usage, especially in clinical settings including in-hospital admission and vascular rehabilitation.
